# eDNAir: proof of concept that animal DNA can be collected from air sampling

**DOI:** 10.7717/peerj.11030

**Published:** 2021-03-31

**Authors:** Elizabeth L. Clare, Chloe K. Economou, Chris G. Faulkes, James D. Gilbert, Frances Bennett, Rosie Drinkwater, Joanne E. Littlefair

**Affiliations:** School of Biological and Chemical Sciences, Queen Mary University of London, London, United Kingdom

**Keywords:** airDNA, eDNA, Biomonitoring, Biodiversity, Terrestrial

## Abstract

Environmental DNA (eDNA) is one of the fastest developing tools for species biomonitoring and ecological research. However, despite substantial interest from research, commercial and regulatory sectors, it has remained primarily a tool for aquatic systems with a small amount of work in substances such as soil, snow and rain. Here we demonstrate that eDNA can be collected from air and used to identify mammals. Our proof of concept successfully demonstrated that eDNA sampled from air contained mixed templates which reflect the species known to be present within a confined space and that this material can be accessed using existing sampling methods. We anticipate this demonstration will initiate a much larger research programme in terrestrial airDNA sampling and that this may rapidly advance biomonitoring approaches. Lastly, we outline these and potential related applications we expect to benefit from this development.

## Introduction

Environmental DNA (eDNA) is DNA which has been shed from sources such as saliva, urine or skin cells and can be accessed from the collection and filtration of non-biological substrates such as water (Ficetola et al., 2008). Both intra- and extra-cellular forms of eDNA are released, giving rise to a continuum of free strands of DNA, mitochondria and intact cells (Sassoubre et al., 2016; Turner et al., 2014). eDNA is increasingly being used for biosurveillance, species occupancy studies, and the detection of endangered and invasive species, particularly in aquatic ecosystems (Cristescu & Hebert, 2018; Deiner et al., 2017). Assay development has focused on single species qPCR for targets of interest, as well as metabarcoding for fish, amphibian, and macroinvertebrate communities (Hänfling et al., 2016; Lacoursière-Roussel et al., 2018; Valentini et al., 2016) and adjacent terrestrial communities which have been detected from pond and lake water (Harper et al., 2019; Ushio et al., 2017), likely through runoff and/or direct interactions with the water source.

Abiotic and biotic factors have been implicated in shaping the release, persistence and degradation of aquatic eDNA which, in turn, shapes the timeframe for detection. Shedding rates, for example, have been found to increase under conditions of higher biomass (Eichmiller, Miller & Sorensen, 2016; Klymus et al., 2015; Takahara et al., 2012), increased temperature (Robson et al., 2016), and under conditions of plentiful food (Klymus et al., 2015). Once released, a key issue which particularly affects detection in riverine habitats is that of signal transport in which community DNA is moved downstream from source populations (Carraro et al., 2018; Deiner & Altermatt, 2014; Deiner et al., 2016; Shogren et al., 2017). However, in contrast to early concerns that DNA would be ubiquitously distributed within aquatic communities and thus show “everything is everywhere”, considerable evidence has been gathered to suggest that DNA is well localised even in fast moving aquatic systems (Harper et al., 2018; Littlefair et al., 2020). Despite these environmental factors, a meta-analysis synthesising 37 studies has demonstrated that eDNA is comparable or exceeds detection compared to conventional methods (in communities under 100 species) in freshwater systems (McElroy et al., 2020).

The collection of eDNA from water has become widely used and the body of literature around the development and application of this method has expanded in the last decade. Indeed, an entire journal (Environmental DNA) has been established for the dedicated study of eDNA. Despite this success, other sources of eDNA e.g., blood, soils, sediments, ice, snow and even honey (Bohmann et al., 2014) have been much less well investigated. For example, soil metabarcoding has been used to detect chordate species in zoos (Andersen et al., 2012), but these techniques have not been extensively deployed for biodiversity surveillance in the same way as aquatic eDNA. Trophic links have also been exploited to detect terrestrial mammals through the use of blood-feeding leeches (e.g., Schnell et al., 2012; Drinkwater et al., 2019) and carrion flies (Calvignac-Spencer et al., 2013), but this relies on the presence and dietary preferences of these hematophages and is not yet being widely used for biomonitoring. 

What is surprising is that no active attempts to collect terrestrial biodiversity from air have been reported, even though reviews on the current state and future direction of eDNA metabarcoding (e.g.,  Ruppert, Kline & Rahman, 2019) mention the next area of interest being airborne DNA mostly in the context of microbes, pollen, and fungi. Early PCR-based studies were used to identify pollen (West et al., 2008; Longhi et al., 2009; Folloni et al., 2012; Mohanty, Buchheim & Levetin, 2017) and a small number of metabarcoding studies of airborne particles have identified anemophilous plants (e.g.,  Johnson, Cox & Barnes, 2019a; Johnson, Cox & Barnes, 2019b), pathogenic microbes (Nicolaisen et al., 2017; Núñez et al., 2017) and allergenic pollen (Kraaijeveld et al., 2015; Korpelainen & Pietilainen, 2017; Núñez et al., 2017; Leontidou et al., 2018; Brennan et al., 2019), with a focus on human and crop health (Yoo et al., 2017). Similarly, Abrego et al. (2018) successfully conducted a fungal biodiversity survey by DNA barcoding fungal spores across Finland, and biodiversity assessments of seasonal plant and fungal diversity have been demonstrated as viable (Banchi et al., 2020). The most common approach has been to use collected dust as a DNA source. For example, Craine et al. (2017) used chloroplast markers to identify plant DNA found in settled dust and demonstrated that geography influenced the distribution of plant matter across the USA. A community analysis constructed of passively collected airborne eDNA identified airborne algae across the Ko’olau mountain range of Hawai’i and found high levels of OTU variation in the algal fingerprint at localised scales, particularly between the windward and leeward side of the range (Sherwood et al., 2017). These results suggest that particulate matter in the air will show spatial–temporal confinement just as eDNA in water has been found to be spatially confined (everything is NOT everywhere). 

Despite the growing interest to date, no viability assessment of animal eDNA carried in air has been published in the scientific literature. An MSc dissertation (Danielsson, 2020) has assessed the viability of identify flying insect species using DNA from air with some success, and high school science fair participants Yuma Okamoto and So Tsukamoto, of Shizuoka Prefectural Kakegawa-Nishi High School in Kanegawa, Japan constructed a bird detector based on airborne DNA and traced the spatial-temporal patterns of DNA degradation. However, these have thus far failed to make their way into the peer-reviewed scientific literature. Here we explore the active collection and filtration of air as a source of mammalian environmental DNA which we term “airDNA” and assess: (1) whether DNA can be extracted from air, (2) whether air volume and filter methods used for aquatic eDNA can be applied directly to airDNA and (3) the source of airDNA collected.

## Materials & Methods

### Sampling procedure

This proof-of-concept study was conducted in a dedicated animal housing room which has contained only naked mole-rats (*Heterocephalus glaber*) for more than a year. *H. glaber* were held in individual artificial burrow systems as social groups (colonies). At the time of sampling there were 225 *H. glaber* individuals in fifteen colonies within the room which was 3 m × 4 m in area. The room also contained some equipment being stored and was accessed frequently by staff who care for the animals. We used a mains powered peristaltic pump (Geotech) attached to Sterivex-HV pressure filters (Merck Millipore). We collected samples “paired” from within the main room with the filter pointing away from the colony and then with the filter inside one artificial burrow, “Colony Omega” which contained 29 individuals within a system of acrylic boxes (3.6 m^3^) connected by tubing 4.4 m in length and 45 mm in internal diameter. For each case we sampled with a 22 µm pore diameter filter and then repeated this with a 45 µm pore diameter filter. For each location, we collected a sample for five, ten or twenty minutes using both filter sizes (twelve samples in total). The pump ran at a 150 ml/min volume and the room was held at a constant temperature between 26−32 °C with a relative humidity of 28–36% during collection. Following sampling, the Sterivex filters were placed in bags and frozen.

The pump used here was specifically selected because it is quiet and when running made less noise than the ambient sounds within the animal room e.g., animal gnawing, human conversation, food preparation. As such the animals were not disturbed by the sound of the pump.

### airDNA extraction

DNA extraction was carried out in a biological safety cabinet under maximum flow. All tools and apparatus had been sterilized using UV and were cleaned in 10% bleach and 70% ethanol between samples. Following Cruaud et al. (2017), the filter was peeled off with sterile forceps and placed in a clean tube. The forceps and pipe cutters used to open the Sterivex filters were sterilised with 50% bleach before use and in between samples, and ultrapure water was used to rinse off the residual bleach. DNA was extracted using a Qiagen DNA Blood and Tissue kit following the manufacturer’s protocol with the following modifications. For all samples the volumes used were: 450 µl ATL buffer (larger volume to ensure all the filter pieces were submerged), 50 µl proteinase K, 500 µl AL buffer and 500 µl 100% ethanol. For negative controls, the reagent volumes were as recommended in the Qiagen handbook. Following overnight lysis and shaking 650 rpm at 56 degrees, the samples were vortexed for fifteen seconds and the liquid transferred to a fresh tube. The filter paper remaining in the original tube was passed through a Qiagen QIA shredder spin column, as per the manufacturer’s instructions, and the flow through collected and pooled with the rest of the sample, after which 500 µl of the AL buffer was added to the sample. Centrifugation steps were as detailed in the Qiagen handbook apart from centrifugation at 11,000 rpm for 3 min following the addition of buffer AW2. The DNA was eluted in 30 µl of elution buffer, which had been pre-heated to 70 °C. The eluent was cycled through the column three times, incubating five minutes each time, to increase the concentration of DNA. Negative controls were extracted along with the sample batch.

### PCR amplification of airDNA

**Figure 1:**
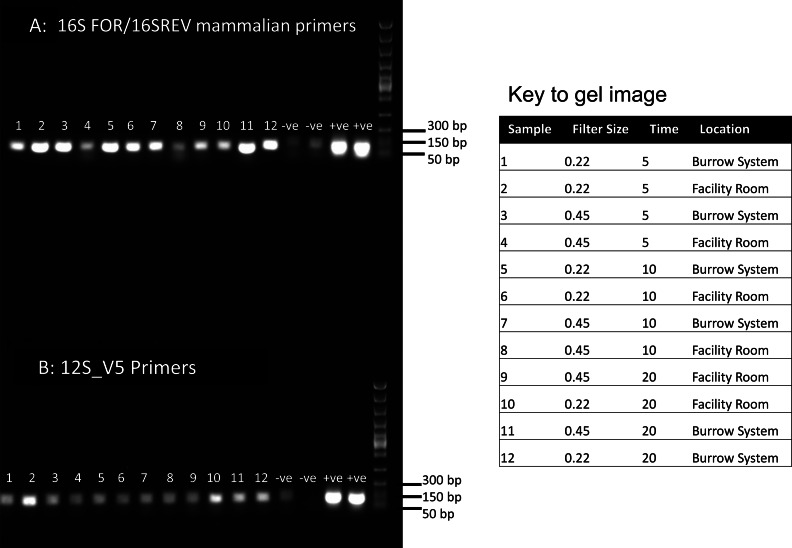
PCR results for filtered air. DNA was extracted from filters and amplified with primers targeting the 16S mitochondrial region (A) using primers designed for mammals (Taylor, 1996) and using vertebrate primers commonly applied in aquatic eDNA (Riaz et al., 2011) for the 12S mitochondrial region (B). In each well 5 µl of PCR reaction was run on a 1% agarose gel. Fast DNA ladder from New England BioLabs was used as a size standard.

Each sample was subjected to PCRs as follows: PCR 1 & 2 included 7.5 µl of Qiagen multiplex mix, 2.5 µl ddH2O, 4 µl of template DNA and 0.5 µl of each forward and reverse primer. We used primers 12S_V5 targeting vertebrate 12S mitochondrial DNA and commonly used for fish eDNA assessment from water (Riaz et al., 2011) and 16S mam1 and 16S mam2 primers targeting mammalian 16S mitochondrial DNA (Taylor, 1996). These PCRs were conducted to compare amplification using two well-known primers targeting different mitochondrial regions. PCR 1&2 amplification conditions were 95 °C for 15 min, then 40 cycles of 94 °C for 30 s, 59 °C for 90 s, 72 °C for 90 s, followed by 72 °C for 10 min and a 10 °C hold. Following assessment of PCR results we proceeded with 16S amplification only, due to poorer amplification of the 12S fragment (Fig. 1). To further assess the DNA content, it was necessary to perform PCRs using modified primers which are compatible with high-throughput sequencing. For PCR 3 we used the 16S primers selected for higher amplification but with the addition of tags appropriate for the Illumina NextSeq platform for high throughput sequencing of mixed templates. In this reaction we used 5 µl of template DNA. To further maximize our sequence recovery, we conducted a nested PCR (PCR4). Nested PCRs can increase amplification success using PCR products rather than DNA as a template, but in mixed diversity samples can risk losing rare DNA (not anticipated here). For PCR 4 we used 0.5 µl of cleaned 16S PCR products as a template in a second PCR reaction using primers tagged for the Illumina NexSeq. For PCR 3&4 the annealing temperature was reduced to 55 °C. All PCRs included extraction negatives and cow DNA as a positive control and products were visualised on a 1% agarose gel.

### Sanger and high-throughput sequencing

From the initial PCR with untagged primers, we selected four products for Sanger sequencing. Samples 3, 4, 6 and 12 from the 16S mammal PCR were cleaned using the Monarch PCR and DNA cleanup kit (New England BioLabs) and 10 ng/µl samples were sequenced at Source Bioscience Ltd, Nottingham UK. The resulting Sanger sequences were edited in CodonCode Aligner and recovered sequences were compared to human and *H. glaber* mitochondrial genomes using BlastN. Tagged PCR amplicons (single and nested PCRs) were quantified using Qubit and Tapestation before addition of Fluidigm tags for pooling samples. The tagged amplicons were sequenced using TSP chemistry on an Illumina NextSeq at the Barts and the London Genome Centre following standard protocols using a unidirectional 75 bp sequencing protocol. The resulting reads were demultiplexed by tags and filtered for the removal of low-quality sequences. We used AdapterRemoval v2 (Schubert, Lindgreen & Orlando, 2016) to remove adapters, and low quality bases and sequences. Primer sequences were then manually removed in Aliview (Larsson, 2014). The remaining reads were processed in the DADA2 pipeline (Callahan et al., 2016) filtering for read quality and clustering into amplicon sequence variants (ASVs) for processing. The ASVs were compared to references in GenBank using BLASTn and we recorded the highest mammal similarity to references for each ASV in each sample. We explored the relationship between the duration of sample filtering time and recovery of mammal sequences to test whether sample filtering time was correlated to data recovery. We used Mann Whitney U tests for independent samples implemented in R v4.0.2 to assess the relative sequence recovery of both mole rat and human sequences using 22 or 45 µm filters.

## Results

### PCR and Sanger sequencing results

**Figure 2:**
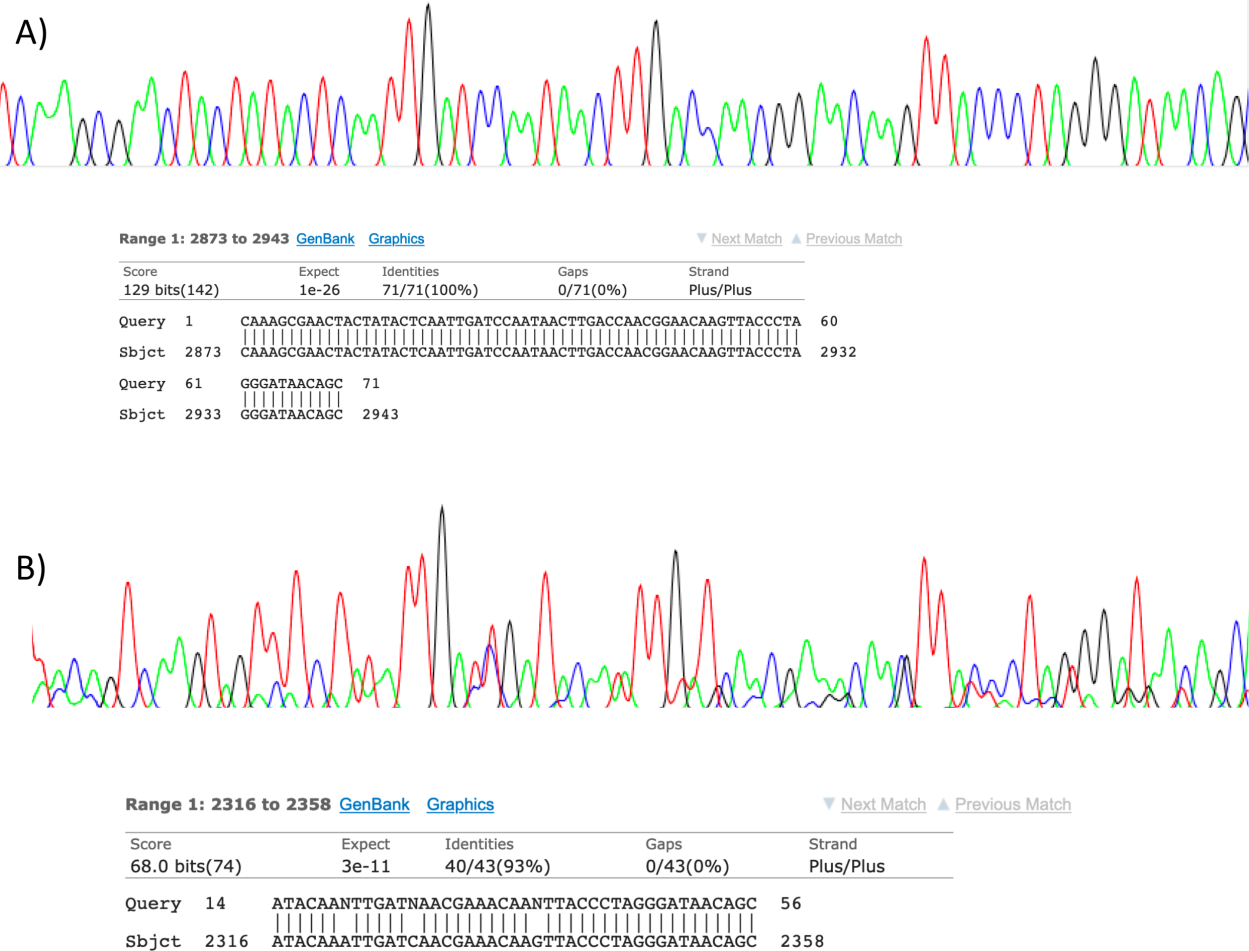
Sanger sequencing assays for DNA collected from air. Clean Sanger sequencing trace (A) of a human sequence and a trace showing mixed templates (B). Below each, the respective BLASTn results of partial templates following editing (query) are compared to existing GenBank reference sequences (sbjct). Unresolved bases (N) in the lower trace preclude a 100% identity match between this fragment and the reference sequence for the naked mole-rat.

Visualisation of PCR results (Fig. 1) demonstrated high amplification success in almost all samples, with brighter bands for 16S mammal specific primers than the 12S primers. As a consequence, we only sequenced 16S products. We observed some background contamination of negative controls. Sanger sequencing of four products produced one clear sequence and three which showed clear evidence of competing DNA signals, expected from mixed template samples. The clear sequence (Fig. 2) was derived from the room and identified with 100% sequence similarity as human. One mixed template sample derived from inside the mole-rat enclosure (sample 3) was edited carefully to remove failed sequence areas and the 3′ portion where the signal showed minimal mixed signal was compared to the nucleotide collection on Genbank resulting in a match to the *H. glaber* mitochondrial genome (Fig. 2). All bases that could be called were identical to those of the *H. glaber* reference.

### High-throughput sequencing results

We recovered sequences for all samples (Table 1) and recorded best mammal BLAST assignments. We removed reads associated with non 16S regions or chromosomes (presumptive NUMTs). We collapsed ASVs and recorded assignments to *H. glaber* (or the related species *Fukomys damarensis* which cannot be easily differentiated at this locus). We also report matches to human and cow (our positive control) and a very small number of 100% matches to dog and sheep. All human matches were >98.5% similar to the reference. The vast majority of mole rat sequences (>96%) were contained in a single ASV and these were assigned to either 100% identical to *H.glaber or F. damerensis* or 98.5% *H. glaber*. Other ASVs were assigned either to *H.glaber* or *F.damerensis* at slightly lower similarity (94.6–98.6%). A small number of sequences were assigned to other rodents but with lower similarity and smaller BLAST bit scores. None of these were in the dominant ASV and were thus excluded. Mole-rat and human were recorded in almost all air samples regardless of collection location, time or filter (mole-rat airDNA detected in 10/12 samples, human airDNA detected in 12/12 samples with 16S primers). Using a nested PCR, mole-rat airDNA was detected in 6/12 samples, five of which were inside the burrow system. No mole-rat DNA was detected in negative controls however human DNA was detected in three negative controls. There was no relationship between the duration of sample filtering time and recovery of mole-rat (*R*^2^ =  − 0.006, *p* = 1.0) or human sequences (*R*^2^ = 0.016, *p* = 0.7) and we saw no effect of filter pore size on the numbers of either mole rat (*W* = 81.5, *p* = 0.597) or human (*W* = 86, *p* = 0.443) sequences obtained.

**Table 1:** High-throughput sequencing outcomes for regular and nested PCRs. Samples consist of the 12 samples described in the collection methods plus two negative and two positive controls. Each was subjected to two PCR procedures (PCR 3&4). Values represent the total number of sequences from each sample assigned to mole-rat and human targets and false positive assignments to dog and sheep, along with sample time, source and filter used to collect the air.

Sample	Sample time	Source	Filter	Mole rat	Human	Cow	Dog	Sheep
1	5	Burrow System	0.22	5,905	836	0	0	0
2	5	Facility Room	0.22	43	12,836	0	0	0
3	5	Burrow System	0.45	6,689	5,196	0	44	0
4	5	Facility Room	0.45	881	5,637	0	344	0
5	10	Burrow System	0.22	4,318	8,734	0	0	0
6	10	Facility Room	0.22	655	8,381	0	1,866	0
7	10	Burrow System	0.45	1,601	2,787	0	0	0
8	10	Facility Room	0.45	530	4,816	0	0	0
9	20	Facility Room	0.45	0	2,534	0	0	0
10	20	Facility Room	0.22	0	8,953	0	0	0
11	20	Burrow System	0.45	8,268	2,255	0	0	0
12	20	Burrow System	0.22	7,756	6,998	0	0	0
Neg 1	NA	NA	NA	0	5,756	0	3,408	0
Neg 2	NA	NA	NA	0	4,186	0	0	0
Pos 1	NA	NA	NA	0	13	21,756	0	91
Pos 2	NA	NA	NA	0	0	16,692	0	96
Nested_1	5	Burrow System	0.22	11,904	7393	0	0	0
Nested_2	5	Facility Room	0.22	0	8,458	0	0	0
Nested_3	5	Burrow System	0.45	10,160	7,561	0	0	0
Nested_4	5	Facility Room	0.45	0	17,713	0	0	0
Nested_5	10	Burrow System	0.22	7,643	11024	0	0	0
Nested_6	10	Facility Room	0.22	0	10,813	0	6,550	0
Nested_7	10	Burrow System	0.45	0	19,363	0	0	0
Nested_8	10	Facility Room	0.45	0	18,289	0	0	0
Nested_9	20	Facility Room	0.45	0	12,219	0	0	0
Nested_10	20	Facility Room	0.22	1,724	10,426	0	674	0
Nested_11	20	Burrow System	0.45	9,075	6,710	0	0	0
Nested_12	20	Burrow System	0.22	7,198	6,786	0	0	0
Nested_Neg 1	NA	NA	NA	0	0	0	0	0
Nested_Neg 2	NA	NA	NA	0	17,146	0	0	0
Nested_Pos 1	NA	NA	NA	0	16	12,390	0	116
Nested_Pos 2	NA	NA	NA	0	65	14,817	0	119

## Discussion

The use of eDNA as a tool in biomonitoring is a rapidly developing and expanding field because of the efficient and non-invasiveness of the approach. Here we demonstrate, for the first time, that air may also be a viable substrate for the collection of environmental DNA from animal life and we provide proof of concept that we can collect, extract and analyse airDNA using readily available tools and analysis pipelines. We recovered mole-rat DNA in both burrow and regular air samples indicating that airDNA can move beyond the confinement of their burrow system. We detected human DNA in all samples, likely reflecting the frequent use of the room by humans. We also detected human DNA contaminating three of our four negative controls which almost certainly reflects the pervasive nature of human DNA in general purpose laboratory facilities. Though many aspects of this approach require refinement we anticipate airDNA will rapidly expand as a data source and we suggest some exciting new applications for airDNA analysis.

### Applications and challenges

Our results suggest that there is strong potential to use air as a source of DNA. We particularly suspect that airDNA will be useful in confined spaces such as manmade structures, tree hollows, caves and subterranean systems where dilution effects may be minimal and mixed species groups are hard to assay visually (e.g., dark or inaccessible). In aquatic systems, the main application of eDNA has been species biomonitoring for spatial presence/absence tracking of individuals, such as invasive species (e.g., Thomas et al., 2020) or the movement of migratory species (e.g., Thalinger et al., 2019) and entire communities. One of the most well-known cases of species monitoring with aquatic eDNA which could act as a model is the detection of the highly protected Great Crested Newt (*Triturus cristatus*) in the United Kingdom (Rees et al., 2014). Extensive regulatory management and intensive visual surveys have been expanded to include eDNA detections (Rees et al., 2017). This provides a good example of how mixed monitoring approaches can be integrated to overcome problems. One potential application where airDNA may be very useful in the terrestrial ecozone is in the survey of species that roost in hollows or caves and are otherwise inaccessible to visual surveys. For example, bats in caves often inhabit areas which are either too small to access or too toxic for safety. In such cases it may be possible to siphon air via a tube to a more accessible location to conduct biosurveys.

One problem in airDNA monitoring may be the dilution effect of large air spaces. It may be that airDNA is particularly useful in confined spaces such as caves, hollows and subterranean systems which replicate our experimental room, but less effective in the open. It is not known whether airDNA may be diluted beyond detection in such cases. In the open air the challenge may be to find a method of vacuuming air at a sufficient rate, while also using a battery-operated device for sampling in remote areas. The pump we used was mains powered but has a battery option which has considerable operational time (hours), but a very low flow rate. Most commercial vacuums have high flow rate but less than an hour of battery life (e.g., 20 min). They are also much noisier, which may be a consideration in the vicinity of live animals.

Conversely, in open settings DNA may be mixed to a meaningless soup, the “everything is everywhere” problem, rather than spatially confined providing a signal for true presence and absence. Similar questions about dilution and mixing were initially raised about large lakes and the ocean, where large volumes and currents could move and dilute DNA until it was no longer useful (Hansen et al., 2018). However, these concerns were largely unfounded and eDNA monitoring has been applied even in the open ocean (Goodwin et al., 2017; Harrison, Sunday & Rogers, 2019). Our data can’t directly address the spatial scale of DNA dilution; however, mole-rat DNA was detected in more samples from within their burrow system than in the main room suggesting that at least some of the DNA signal is localized to the burrow.

While not our target, most air samples contained human DNA, suggesting forensic applications. DNA has been employed in criminal analysis for nearly 30 years (Royal Society of Edinburgh, 2017) with short tandem repeats (STRs), Y-chromosome and mitochondrial DNA as common targets. More advanced sequencing approaches have started to gain traction, such as targeted enrichment of highly degraded mitochondrial DNA (Young, Higgins & Austin, 2019) which has aided in the identification of matrilineal associations (e.g., skeletal remains). We targeted 12S or 16S fragments as a simpler approach for proof of concept, but in combination with targeted enrichment, metagenomic approaches might be viable for recovering longer DNA fragments or STRs used in conventional DNA profiling. Using air as the source may make it possible to recover forensic traces of recent activity even when no physical traces (such as blood or hair) have been left, for non-invasive DNA collection or in forensic anthropology. While our detection of human DNA was not surprising given the enclosed environment and the frequent use of the space by humans, it highlights the potential of air as a DNA source in forensics but that it may also represent a contaminant which may swamp target trace sources. Blocking probes may be needed if human DNA is ubiquitous. It is possible that the human DNA is in higher concentration when compared with the signal from animals, but it is equally possible that the primers preferentially bind to human DNA and thus we see more amplification. Quantification from such data is highly controversial and should be treated with caution (Deagle et al., 2019), thus we will not speculate further.

One interesting potential application is determining the mechanisms of airborne pathogen transmission. In the current pandemic, international policies (Mahase, 2020) and economies hang on determining “social distance” (Lewnard & Lo, 2020) which is now part of both the public conversation and scientific literature (e.g., Bourouiba, 2020). The key is determining what concentration of viable viral particles is sufficient to constitute an infectious dose and over what distance that volume can be delivered. Viral particles and RNA can be collected using a similar approach to the method we have suggested here and with refinements in regard to quantification of a specific target, it may be possible to better define and apply safety guidelines. It is interesting that we saw no effect of filter size, though our sample is small, and this requires further testing. It may be that filter size will actually help target different taxa e.g., whole microbes or pollen spores vs. DNA fragments.

The filter we used is similar to a HEPA filter. Most homes in the industrialised world employ HEPA filters (e.g., heating, ventilation) potentially collecting eDNA as a byproduct of regular operation. There is a very strong potential that such filters could be collected as part of a monitoring scheme, potentially for targets such as pests (mice, rats, termites, cockroaches) inside the home or birds or insects outside, though again blocking probes would be useful to limit the overwhelming signal from human/mammalian pets in homes. One interesting idea is to couple this technology with other bio-monitoring approaches such as acoustic sampling, camera trapping, soil eDNA and GPS/GIS for multi-modal monitoring approaches, which are as exciting as they are challenging to coordinate.

### Validation and future work

Before any application can be considered it is necessary to consider the approaches which should be used for validation. As part of the development of aquatic eDNA tools across Europe a validation scale has been established (https://edna-validation.com/) which sets out a series of five stages of validation for the detection of a species of interest. This scale is slightly limited as some categories are not applicable to community/biodiversity testing, but with some modifications the discrete steps that should be passed before moving to the next stage of validation are a very useful guide. Our basic proof of concept meets the first stage criteria (*in silico* analysis and initial PCR) and the second stage criteria (PCRs are more optimised, *in vitro* testing and validated detection of eDNA target from laboratory environments). We suggest we have demonstrated a stage two validation of air as a viable source of mammal DNA. The next most important steps are to conduct extensive field-based testing and determine the factors which may limit the use of this tool. In particular we need to determine the limits of detectability which will include assessing the spatial scale of airDNA detection and the environmental factors which contribute to airDNA degradation but should also consider factors such as differences in target species biology (biomass, fur type) and air sample volume. Most fundamentally the next step is to expand the assessment in terms of taxa, spatial scale and sample size, permitting much more specific hypothesis testing of factors which will contribute to the detection of target DNA sources. Still, we suggest that this proof of concept should be considered as a guide for further developments and we anticipate the rapid expansion of this novel field of analysis.

## Conclusions

Our analysis provides a first proof of concept demonstration that air samples are a viable source of DNA for the identification of species in the environment. We demonstrate that even short sampling times (e.g., five min) using low powered air collection can produce positive PCR and sequencing outcomes for multiple target species. Following the success of eDNA sampling from water, we anticipate that sampling for airDNA will become useful in a wide variety of non-invasive applications from ecological sampling to forensic analysis.

## Additional Information and Declarations

### DNA Deposition

The following information was supplied regarding the deposition of DNA sequences: Sequence data are available at Figshare: Clare, Elizabeth (2021): eDNAir: proof of concept that animal DNA can be collected from air sampling.. figshare. Dataset. https://doi.org/10.6084/m9.figshare.13168298.v1.

### Data Availability

The following information was supplied regarding data availability: Sequence data are available at Figshare: Clare, Elizabeth (2021): eDNAir: proof of concept that animal DNA can be collected from air sampling.. figshare. Dataset. https://doi.org/10.6084/m9.figshare.13168298.v1.
